# Person‐Centered Care, Care Climate, and Quality of Life: Evidence From Inpatient Settings in Kazakhstan

**DOI:** 10.1155/jonm/2533736

**Published:** 2026-10-21

**Authors:** Amirshair Abdilda, Joseph Almazan, Gulnur Nadirbekova, Jonas Preposi Cruz

**Affiliations:** ^1^ Department of Medicine School of Medicine Nazarbayev University Kerey and Zhanibek Khans St 5/1 Astana 010000 Kazakhstan nu.edu.kz; ^2^ Corporate Fund University Medical Center Astana Kazakhstan

**Keywords:** care climate, Kazakhstan, nursing management, patient experience, person-centered care, quality of life

## Abstract

**Background:**

Person‐centered care (PCC) is recognized as a core indicator of high‐quality nursing and organizational performance. Although PCC and supportive care climates have been associated with patient outcomes, limited evidence exists from Central Asia on how these factors relate to patients’ quality of life (QOL).

**Aim:**

To examine associations between adult inpatients’ perceptions of PCC, the care climate, and their QOL in Kazakhstan.

**Design:**

Cross‐sectional analytical study.

**Methods:**

A total of 255 adult inpatients from two public hospitals completed the Patient‐Centered Inpatient Scale (P‐CIS), the Person‐Centered Climate Questionnaire–Patient version (PCQ‐P), and the WHOQOL‐BREF. Multivariable linear regression was used to examine associations between PCC, care climate, sociodemographic variables, and QOL across physical, psychological, social, and environmental domains.

**Results:**

In the adjusted models, overall PCC experience was associated with psychological, social relationships, and environmental QOL, while overall PCC climate was associated with physical and psychological QOL. Across the four models, *R* ^2^ values ranged from 0.192 to 0.328, with adjusted *R* ^2^ values ranging from 0.120 to 0.267. In the bivariate analyses, several individual PCC and care‐climate dimensions, including empowerment, safety, and information provision, were associated with specific QOL domains; however, these dimensions were not entered separately into the multivariable models. Participants generally reported positive experiences of respectful and supportive care. PCC‐related measures also varied across several sociodemographic characteristics, suggesting differences in reported care experiences across participant groups.

**Conclusions:**

Overall PCC experience and PCC climate were associated with selected QOL domains after adjustment, while associations involving individual PCC dimensions were observed only in bivariate analyses and should therefore be interpreted cautiously. Given the exploratory nature of the analyses, the absence of formal adjustment for multiple comparisons, and the modest explanatory power of the adjusted models, the findings should not be interpreted as evidence of independent or causal effects. Communication, patient involvement, and culturally responsive care remain important considerations for nursing management. However, patients’ QOL is likely shaped by a broader combination of clinical, social, and contextual factors beyond PCC alone.

**Implications for Nursing Management:**

Nurse managers play a critical role in shaping care environments that support empowerment, cultural responsiveness, and effective information exchange. Strengthening communication systems, ensuring linguistic and cultural accessibility, and supporting relational competence among staff may help strengthen PCC and patient‐reported care experiences.

**Highlights**
• What is already known about this topic?
◦ PCC and supportive care climates are established indicators of high‐quality nursing and organizational performance, contributing to improved patient experiences and system outcomes.
◦ Relational elements of care, particularly communication, empowerment, and perceived safety, are important features of patient experience across diverse healthcare environments.
◦ Despite national reforms promoting people‐centered care, there is limited empirical evidence from Central Asia examining how PCC and care climate relate to patients’ quality of life (QOL).
• What this paper adds?
◦ Overall PCC experience and PCC climate were associated with selected QOL domains after adjustment, with associations varying across physical, psychological, social, and environmental QOL.
◦ Individual PCC and care‐climate dimensions, including empowerment and perceived safety, were associated with specific QOL domains in bivariate analyses, highlighting potentially relevant aspects of care for further investigation.
◦ Sociodemographic differences, especially language proficiency and socioeconomic status, were associated with PCC perceptions, emphasizing the necessity for nurse managers to strengthen culturally responsive communication and support equitable organizational practices within hospital settings.

## 1. Introduction

Healthcare systems worldwide have gradually shifted from a predominantly biomedical orientation toward models of care that give greater attention to psychosocial well‐being, patient experience, and the everyday conditions in which care is delivered. This transition reflects a broader understanding of healthcare as supporting dignity, participation, functioning, and well‐being, rather than disease management alone [1]. Within this context, person‐centered care (PCC) has become a central approach for strengthening the quality of care and the way services are organized around patients’ needs. PCC emphasizes patients’ values, preferences, capabilities, and experiences and supports care processes grounded in dignity, partnership, and meaningful involvement in decision‐making [2, 3]. Previous studies have associated PCC with better patient experience, stronger therapeutic relationships, greater satisfaction, perceived care quality, and QOL outcomes [4–6].

International research has increasingly linked PCC with care quality, patient satisfaction, care coordination, and organizational performance. In settings where PCC principles are embedded in routine practice, studies have reported shorter hospital stays, fewer readmissions, and stronger coordination of care [3, 4]. These outcomes, however, are unlikely to depend on isolated clinical interactions alone. They are also shaped by how care is organized; how communication occurs; and whether patients experience the care environment as safe, respectful, and responsive. Evidence from hospital and long‐term care settings suggests that patients’ experiences of PCC are closely connected to communication, involvement, respect, safety, hospitality, and satisfaction [5, 7, 8]. Research from Australia [9], Spain [6], Iran [7], and Sweden [10] and other healthcare contexts further suggests that PCC is relevant to patient engagement, empowerment, and psychosocial well‐being, although these associations may vary across cultural, clinical, and organizational settings. Thus, this evidence indicates that PCC should be understood not only as a set of interpersonal care practices but also as part of the broader care climate experienced by patients during hospitalization.

Kazakhstan has undertaken substantial health system reforms aimed at shifting from a centralized, hospital‐oriented model toward more socially oriented and people‐centered care [1, 11]. Despite these reforms, the implementation of PCC remains uneven. Despite limitations in patient‐centered communication and responsiveness to patient preferences, inpatient settings have reported high levels of patient satisfaction [12]. More recent national evidence from primary healthcare similarly indicates moderate patient‐centered performance, with considerable regional and health‐system variation [13]. Collectively, this evidence suggests that patient experiences in Kazakhstan reflect not only interpersonal aspects of care but also organizational responsiveness and broader health‐system conditions. Within inpatient services, nurses are particularly important because of their sustained contact with patients and their role in communication, care coordination, and patient involvement. However, organizational and workforce conditions may constrain opportunities for nurses to provide consistently person‐centered and relational care. From a nursing management perspective, addressing these conditions is important for strengthening the care environment and supporting more responsive and patient‐centered service delivery.

Self‐determination theory was used as the primary conceptual lens for interpreting the relationship between PCC, care climate, and QOL. The theory proposes that well‐being is supported when individuals experience autonomy, competence, and relatedness [14]. These needs align with central features of person‐centered inpatient care, including involvement in decisions, access to understandable information, and respectful interactions with healthcare staff [2, 3]. Autonomy is reflected in patients’ opportunities to participate in decisions about their care. Competence is supported when patients receive information that helps them understand their condition, treatment, and care processes. Relatedness is reflected in respectful, responsive, and supportive interactions with nurses and other healthcare staff [14]. From this perspective, PCC and a supportive care climate are understood as relational and organizational conditions associated with patient‐reported QOL during hospitalization [5, 8]. In this study, empowerment, perceived safety, communication, and social support are treated as related interpretive concepts rather than separate theoretical frameworks. This keeps the theoretical framing focused and helps ensure that the interpretation remains close to the study findings. Given the cross‐sectional design, the framework is used to guide interpretation of associations, not to suggest causal mechanisms or psychological pathways.

Despite ongoing reforms toward more people‐centered care, research in Kazakhstan has focused largely on primary healthcare performance and patient satisfaction, while evidence examining person‐centered inpatient care, perceived care climate, and multidimensional QOL within the same study remains limited [12, 13]. In particular, little is known about whether patients’ perceptions of PCC and the broader care climate are associated with different physical, psychological, social, and environmental dimensions of QOL during hospitalization. Examining both PCC experience and PCC climate is relevant because they capture related but distinct aspects of care: patients’ direct experiences of person‐centered interactions and their perceptions of the wider environment in which care is delivered. This distinction is particularly relevant to nursing management because communication, information provision, patient involvement, and the organization of relational care are areas that may be influenced by nursing practice and service organization. By examining PCC experience, PCC climate, and multidimensional QOL within the same inpatient sample, this study extends the Kazakhstan evidence base beyond conventional measures of patient satisfaction and provides context‐specific evidence relevant to nursing management. This study examined the associations between patients’ perceptions of PCC, PCC climate, and QOL among hospitalized adults in Kazakhstan.

## 2. Methods

### 2.1. Aim

This study examined the associations between adult inpatients’ perceptions of PCC, the broader PCC climate within hospital settings, and their overall QOL.

### 2.2. Design

This study used a quantitative, cross‐sectional correlational design and was reported in accordance with the STROBE guidelines for observational studies.

### 2.3. Samples and Settings

The study was conducted in two specialized inpatient hospitals within a large academic medical organization in Kazakhstan. To maintain anonymity, the sites are referred to as Hospital A and Hospital B. Both hospitals provide specialized inpatient care and follow internationally recognized standards for quality and patient safety.

The study included adult patients receiving inpatient care at either hospital. The inpatient setting was selected because hospitalization provides patients with sustained exposure to interpersonal interactions, care processes, and the broader care environment, allowing them to evaluate both PCC and the perceived care climate. Data were collected near the time of discharge, after participants had experienced most of their hospitalization, enabling them to reflect on their care experience while minimizing the potential for recall bias associated with assessments made after discharge.

The inclusion criteria were (1) patients who had stayed at least 24 h in either of the two hospitals; (2) patients receiving direct care from nurses and physicians; (3) patients about to be discharged; (4) patients aged 18–64 years; (5) patients competent in Kazakh or Russian; and (6) patients who were conscious, coherent, and able to engage in conversation. Patients were excluded if they had health conditions that prevented accurate survey completion (e.g., severe illness, cognitive impairment, delirium, communication disorders, or extreme fatigue/pain). Pediatric patients and those older than 64 years were also excluded.

Convenience sampling was used. The sample size was calculated a priori for standard multiple regression using G∗Power Version 3.1 [15]. With 21 predictor variables, a medium effect size (*f* ^2^ = 0.15), 90.0% statistical power, and a 0.05 level of significance, the required sample size was 195 participants. After allowing for a potential 20% nonresponse or attrition rate, the target sample size was 234 participants.

### 2.4. Instruments

Data were collected using a structured survey composed of a sociodemographic questionnaire and three standardized instruments: the Patient‐Centered Inpatient Scale (P‐CIS), the PCC Climate Questionnaire–Patient version (PCQ‐P), and the World Health Organization QOL–BREF (WHOQOL‐BREF). The sociodemographic questionnaire included age, gender, religion, area of residence, education, employment status, marital status, financial status, and language proficiency.

The P‐CIS was used to assess patients’ perceptions of person‐centered inpatient care. The instrument includes 20 items. In the present analysis, item scores were summarized using the following labeled groupings reflected in the dataset: personalization, empowerment, information, approachability/availability, respectfulness, and miscellaneous [16, 17]. Responses are rated on a 5‐point Likert scale, with higher scores indicating more positive perceptions of PCC. Negatively worded items were reverse‐coded before computing the mean score. The original scale demonstrated good reliability, with a Cronbach’s alpha of 0.91 [17]. Permission to use the instrument was obtained from Professor Brian Williams via email (B. Williams, personal communication, September 28, 2024).

The PCQ‐P was used to assess patients’ perceptions of the PCC climate. The scale consists of 17 items covering three dimensions: safety, everydayness, and hospitality [2, 8]. Responses are rated on a 6‐point Likert scale ranging from 1 (*no, I disagree completely*) to 6 (*yes, I agree completely*). Higher scores indicate a more positive perception of the care climate. The scale has demonstrated satisfactory internal consistency, with a Cronbach’s alpha of 0.89 [8]. Permission to use the instrument was obtained from Professor David Edvardsson via email (D. Edvardsson, personal communication, October 10, 2024).

The WHOQOL‐BREF was used to assess QOL. It contains 26 items covering four domains: physical health, psychological health, social relationships, and environment, together with two general items on overall QOL and health satisfaction [18, 19]. Items are rated on a 5‐point Likert scale. Negatively worded items were reverse‐coded, and higher scores indicated better perceived QOL. The Russian version has shown good reliability (Cronbach’s alpha = 0.842), while the Kazakh version has demonstrated acceptable internal consistency (Cronbach’s alpha = 0.73) [20, 21]. Permission to use the WHOQOL‐BREF was granted by the WHO Permission Team (personal communication, September 23, 2024).

### 2.5. Linguistic Adaptation

The Kazakh and Russian versions of the P‐CIS and PCQ‐P were prepared using forward–backward translation based on the cross‐cultural adaptation process described by Beaton et al. [22]. The process included forward translation, synthesis, backward translation, expert committee review, and pilot testing.

Five expert nurses reviewed the translated questionnaires for relevance and clarity. Each item was rated on a 4‐point relevance scale, and item‐level and scale‐level content validity indices were calculated [23]. All items in both Kazakh and Russian versions received an I‐CVI of 1.00, and the S‐CVI/Ave was also 1.00 for both language versions, indicating strong content validity.

Pilot testing was conducted with 30 patients for each language version. Participants completed the questionnaires and provided feedback on item clarity and readability. Internal consistency was also assessed during pilot testing. In the main study, the P‐CIS showed acceptable reliability in both Kazakh (*α* = 0.80) and Russian (*α* = 0.85). The PCQ‐P demonstrated excellent reliability in Kazakh (*α* = 0.96) and Russian (*α* = 0.97). The WHOQOL‐BREF also showed satisfactory overall reliability in Kazakh (*α* = 0.86) and Russian (*α* = 0.87).

### 2.6. Ethical Considerations

Ethical approval was obtained from the Nazarbayev University Institutional Research Ethics Committee (NU‐IREC) (Application No. 986/05112024; approved 27 January 2025) and the Local Bioethics Committee of the Corporate Fund University Medical Center (Ref. No. 2025/03‐004). Both institutional approvals were obtained before data collection commenced on 17 February 2025. All participants received information about the study purpose, procedures, voluntary nature of participation, and their right to withdraw without consequence. Written informed consent was obtained from each participant before data collection. No personal identifiers were collected, and study data were stored in password‐protected files accessible only to the research team.

### 2.7. Data Collection

Data were collected between February 17 and March 10, 2025, using paper‐based questionnaires. Patients were recruited through face‐to‐face interactions, during which the study objectives and procedures were explained, and written informed consent was obtained. Participants were given adequate time to ask questions and decide on participation. Following consent, the questionnaires were distributed and completed by patients at their own pace, without researcher interference, to minimize bias. Completed forms were collected immediately, with confidentiality and secure data handling strictly maintained throughout the process.

### 2.8. Data Analysis

Data were analyzed using IBM SPSS Statistics, Version 24.0. Descriptive statistics, including frequencies, means, and standard deviations, were used to summarize participant characteristics and study variables. The distributions of the P‐CIS and PCQ‐P scores were non‐normal; therefore, nonparametric methods were used for the bivariate analyses involving these measures. Spearman’s rank correlation coefficient (*ρ*), the Mann–Whitney U test, and the Kruskal–Wallis test were used, as appropriate, to examine associations between sociodemographic characteristics, PCC experience, and PCC climate. Spearman’s rank correlation coefficient was also used to examine bivariate associations among PCC experience, PCC climate, and the QOL domains.

Multiple linear regression analyses were conducted to examine the adjusted associations of sociodemographic characteristics, overall PCC experience, and overall PCC climate with QOL. Four separate regression models were estimated, with physical, psychological, social relationships, and environmental QOL entered individually as the dependent variable. Overall PCC experience and overall PCC climate were included as the principal independent variables together with the sociodemographic covariates entered in each model. Individual dimensions of the P‐CIS and PCQ‐P were not entered separately into the multivariable models; therefore, dimension‐level findings represent bivariate associations only. Categorical variables with more than two categories, including religion, education level, employment status, marital status, and financial status, were dummy coded before inclusion in the regression models.

Before model estimation, the assumptions of multiple linear regression were assessed. Linearity and homoscedasticity were examined using plots of residuals against predicted values, while normality of residuals was assessed using histograms and Q–Q plots. Multicollinearity was evaluated using tolerance and variance inflation factor (VIF) values, with tolerance values below 0.10 and VIF values above 10 considered indicative of potential multicollinearity. No substantial violations of these assumptions were identified.

All questionnaires were checked for completeness at the point of collection. Complete data were available for all 255 participants across the variables included in the descriptive, bivariate, and regression analyses. No item‐level or variable‐level missing data were identified; therefore, all analyses used the full sample (*n* = 255), with no imputation or case exclusion for missing data required.

The bivariate and regression analyses were exploratory rather than confirmatory. Given the number of associations examined across the four QOL domains, no formal adjustment for multiple comparisons was applied. Findings with *p* values close to 0.05 were therefore interpreted cautiously and considered hypothesis‐generating rather than definitive. Statistical significance was set at *p* < 0.05. For each regression model, Table 1 reports the unstandardized regression coefficients (*B*) with 95% confidence intervals, *R* ^2^, adjusted *R* ^2^, the F statistic with its degrees of freedom, and the overall model *p* value.

**TABLE 1:** Multivariable linear regression analyses of quality‐of‐life domains (*n* = 255).

Predictor	Physical health B [95% CI], *p*	Psychological health B [95% CI], *p*	Social relationships B [95% CI], *p*	Environmental health B [95% CI], *p*
*Demographics*				
Age	−0.31 [−0.48, −0.13] *p* = 0.001^∗∗^	−0.07 [−0.23, 0.09] *p* = 0.374	−0.25 [−0.45, −0.06] *p* = 0.012^∗^	−0.13 [−0.31, 0.04] *p* = 0.143
Gender	−2.61 [−7.16, 1.95] *p* = 0.260	−0.87 [−4.86, 3.11] *p* = 0.667	−1.48 [−6.48, 3.52] *p* = 0.561	1.17 [−3.32, 5.65] *p* = 0.609
Christianity (vs. Islam)	3.34 [−4.79, 11.46] *p* = 0.420	3.09 [−4.03, 10.20] *p* = 0.394	10.54 [1.62, 19.47] *p* = 0.021^∗^	1.30 [−6.71, 9.31] *p* = 0.750
Nontheistic (vs. Islam)	0.96 [−6.89, 8.81] *p* = 0.810	−1.65 [−8.51, 5.22] *p* = 0.637	2.71 [−5.91, 11.32] *p* = 0.536	−4.25 [−11.98, 3.48] *p* = 0.280
Hospital	1.09 [−3.86, 6.05] *p* = 0.664	−3.42 [−7.76, 0.92] *p* = 0.122	−1.69 [−7.13, 3.75] *p* = 0.542	0.15 [−4.73, 5.04] *p* = 0.951
Area of residence (urban)	−1.90 [−6.44, 2.65] *p* = 0.412	0.14 [−3.83, 4.12] *p* = 0.944	2.18 [−2.81, 7.17] *p* = 0.390	6.02 [1.55, 10.50] *p* = 0.009^∗∗^

*Education (ref: high school or lower)*				
College graduate	0.66 [−5.26, 6.58] *p* = 0.826	−2.85 [−8.04, 2.33] *p* = 0.279	4.15 [−2.36, 10.65] *p* = 0.210	−2.72 [−8.56, 3.12] *p* = 0.359
University graduate (BA)	4.98 [−0.98, 10.94] *p* = 0.101	−0.17 [−5.39, 5.04] *p* = 0.948	0.29 [−6.26, 6.83] *p* = 0.932	−0.18 [−6.05, 5.69] *p* = 0.951
University graduate (MA/PhD)	3.20 [−4.40, 10.80] *p* = 0.408	−4.88 [−11.53, 1.77] *p* = 0.149	1.22 [−7.12, 9.56] *p* = 0.774	−2.57 [−10.05, 4.92] *p* = 0.500

*Occupation (ref: employed)*				
Unemployed	3.26 [−1.93, 8.46] *p* = 0.217	−2.11 [−6.65, 2.44] *p* = 0.362	0.77 [−4.93, 6.47] *p* = 0.790	1.96 [−3.15, 7.08] *p* = 0.451
Self‐employed	0.77 [−4.60, 6.14] *p* = 0.777	−4.30 [−9.00, 0.40] *p* = 0.073	0.71 [−5.18, 6.60] *p* = 0.812	−3.05 [−8.34, 2.24] *p* = 0.257
Retired	0.69 [−5.20, 6.58] *p* = 0.817	1.34 [−3.82, 6.49] *p* = 0.610	0.89 [−5.58, 7.36] *p* = 0.786	2.03 [−3.78, 7.83] *p* = 0.492

*Marital status (ref: married)*				
Single	1.59 [−4.94, 8.12] *p* = 0.632	2.73 [−2.98, 8.45] *p* = 0.347	−3.63 [−10.79, 3.54] *p* = 0.320	3.67 [−2.77, 10.10] *p* = 0.263
Other (cohabiting/divorced/widowed)	−2.14 [−7.80, 3.51] *p* = 0.456	−0.11 [−5.06, 4.84] *p* = 0.964	−8.45 [−14.65, −2.24] *p* = 0.008^∗∗^	−5.71 [−11.29, −0.14] *p* = 0.044^∗^

*Financial status (ref: < 250 K KZT)*				
250–499 K	0.54 [−4.13, 5.22] *p* = 0.819	1.89 [−2.20, 5.98] *p* = 0.364	1.13 [−4.00, 6.26] *p* = 0.665	4.41 [−0.19, 9.02] *p* = 0.060
500–749 K	2.89 [−2.90, 8.68] *p* = 0.326	−1.09 [−6.16, 3.98] *p* = 0.673	0.18 [−6.18, 6.53] *p* = 0.956	3.85 [−1.85, 9.56] *p* = 0.185
≥ 750 K	−0.18 [−7.29, 6.92] *p* = 0.959	−0.60 [−6.81, 5.62] *p* = 0.850	0.44 [−7.36, 8.24] *p* = 0.911	7.21 [0.21, 14.20] *p* = 0.044^∗^

*Language proficiency*				
Kazakh	1.12 [0.31, 1.93] *p* = 0.007^∗∗^	1.05 [0.35, 1.76] *p* = 0.004^∗∗^	1.48 [0.59, 2.37] *p* = 0.001^∗∗^	1.49 [0.69, 2.28] *p* < 0.001^∗∗∗^
Russian	0.02 [−0.74, 0.77] *p* = 0.964	0.27 [−0.39, 0.94] *p* = 0.415	−0.17 [−1.00, 0.66] *p* = 0.686	−0.20 [−0.94, 0.55] *p* = 0.598

*Person-centered care*				
PCC experience (overall)	2.33 [−1.35, 6.01] *p* = 0.213	3.27 [0.05, 6.49] *p* = 0.047^∗^	4.67 [0.63, 8.71] *p* = 0.024^∗^	3.82 [0.19, 7.44] *p* = 0.039^∗^
PCC climate (overall)	2.00 [0.14, 3.86] *p* = 0.035^∗^	1.90 [0.27, 3.53] *p* = 0.022^∗^	1.17 [−0.87, 3.21] *p* = 0.258	1.76 [−0.07, 3.59] *p* = 0.059

*R* ^2^	**0.215**	**0.192**	**0.231**	**0.328**

Adjusted *R* ^2^	**0.145**	**0.120**	**0.162**	**0.267**

F (21, 233)	**3.05**	**2.64**	**3.34**	**5.41**

Model *p*	**< 0.001**	**< 0.001**	**< 0.001**	**< 0.001**

*Note:* Values are unstandardized regression coefficients (B) with 95% confidence intervals in brackets and two‐sided *p* values. Reference categories are shown in parentheses. *R* ^2^, adjusted *R* ^2^, F statistics, confidence intervals, and *p* values reproduce the values recorded in the original capstone regression tables. Bold values represent the overall regression model statistics (*R* ^2^, adjusted *R* ^2^, *F* statistics, and model *p* values) and do not independently indicate statistical significance. Statistical significance is indicated by the asterisks defined in the table note.

^∗^ *p* < 0.05.

^∗∗^ *p* < 0.01.

^∗∗∗^ *p* < 0.001.

## 3. Results

### 3.1. Sociodemographic Characteristics of the Respondents

A total of 261 patients were approached, of whom 255 agreed to participate, yielding a response rate of 97.7%. Participants ranged in age from 19 to 64 years (*M* = 43.67, SD = 12.94). On a 0–10 self‐rated proficiency scale, mean language proficiency scores were 7.87 (SD = 3.09) for Kazakh and 8.19 (SD = 2.45) for Russian. Of the participants, 61.2% were female, and 38.8% were male. Most participants identified as Muslim (83.5%), while 10.2% identified as Christian and 6.3% reported nontheistic beliefs. Additional sociodemographic characteristics are presented in Table 2.

**TABLE 2:** Demographic characteristics of the respondents (*n* = 255).

**Characteristics**	**Range**	**Mean (SD)**

Age	19.00–64.00	43.67 (12.94)
Kazakh language proficiency	0.00–10.00	7.87 (3.09)
Russian language proficiency	0.00–10.00	8.19 (2.45)

	**n**	**%**

Gender		
Male	99	38.8
Female	156	61.2
Religion		
Islam	213	83.5
Christianity	26	10.2
Nontheistic (atheist, agnostic, and no	16	6.3
religion)		
Hospital		
Hospital A	97	38.0
Hospital B	158	62.0
Area of residence		
Rural area	50	19.6
Urban area	205	80.4
Education		
High school graduate and lower	31	12.2
College graduate	80	31.4
University graduate (bachelor’s degree)	114	44.7
University graduate (master’s or doctorate)	30	11.8
Occupation		
Employed	155	60.8
Unemployed	37	14.5
Self‐employed	31	12.2
Retired	32	12.5
Marital status		
Single	21	8.2
Married	207	81.2
Others (cohabiting, divorced, and widowed)^a^	27	10.6
Financial status		
Below 250,000 KZT	88	34.5
250,000–499,999 KZT	92	36.1
500,000–749,999 KZT	46	18.0
750,000 KZT and above	29	11.4

*Note:* Cohabiting = 9, divorced = 15, widowed = 3.

Most respondents were recruited from Hospital B (62.0%), while 38.0% were recruited from Hospital A. Educational attainment varied, with 12.2% having high school education or lower, 31.4% being college graduates, 44.7% holding a bachelor’s degree, and 11.8% possessing a master’s or doctorate. For employment status, 60.8% were employed, 14.5% unemployed, 12.2% self‐employed, and 12.5% retired. The respondents were predominantly married (81.2%), with 8.2% single and 10.6% classified as cohabiting, divorced, or widowed. Financial status was distributed with 34.5% earning below 250,000 KZT, 36.1% between 250,000 and 499,999 KZT, 18.0% between 500,000 and 749,999 KZT, and 11.4% earning 750,000 KZT or above (Table 2).

### 3.2. Perceived PCC Experience at University Medical Center and Associated Demographic Variables

The overall P‐CIS score ranged from 2.30 to 5.00, with a mean of 3.82 (SD = 0.52). Among the six item groupings, empowerment had the highest mean score (*M* = 4.09, SD = 0.66), while information was rated the lowest (*M* = 3.44, SD = 0.84). Item‐level analysis showed variation across the scale. The highest‐rated item was “The doctors understood fully what I was going through” (*M* = 4.40), while “I wish the doctor had given me a fuller explanation of my condition” had the lowest mean score (*M* = 2.67) (Supporting Table 1).

The bivariate analyses showed a weak negative association between age and PCC experience (*ρ* = −0.17, *p* = 0.007). Participants residing in rural areas reported lower PCC experience scores than those residing in urban areas (*U* = 3615.00, *p* = 0.001). PCC experience also differed significantly across marital‐status groups (*H* = 6.83, *p* = 0.033). Financial status was weakly negatively associated with PCC experience (*ρ* = −0.20, *p* = 0.001). No statistically significant associations were observed between PCC experience and sex, religion, hospital site, education level, employment status, or language proficiency (Table 3).

**TABLE 3:** Results of the bivariate analyses between the respondents’ demographic variables and person‐centered care experience in the hospital (*n* = 255).

Variable	Mean rank	Statistical test	*p*
Age		*ρ = *−0.17	0.007^∗∗^
Gender			
Male	124.55	*U = *7380.00	0.551
Female	130.19		
Religion			
Islam	130.07	*H = *3.28	0.192
Christianity	130.96		
Nontheistic (atheist, agnostic, and no religion)	95.69		
Hospital			
Hospital A	136.25	*U = *6862.50	0.161
Hospital B	122.93		
Area of residence			
Rural area	97.80	*U = *3615.00	0.001^∗∗^
Urban area	135.37		
Education		*ρ* = 0.11	0.073
Occupation			
Employed	121.33	*H = *3.47	0.325
Unemployed	142.62		
Self‐employed	134.19		
Retired	137.41		
Marital status^a^			
Single	149.17	*H = *6.83	0.033^∗^
Married	129.97		
Others (cohabiting, divorced, and widowed)^a^	96.44		
Financial status		*ρ* = −0.20	0.001^∗∗^
Kazakh language proficiency		*ρ* = 0.09	0.170
Russian language proficiency		*ρ* = 0.08	0.203

^a^Single versus others (*U = *179.50, *p* = 0.031) and married versus others (*U = *2046.50, *p* = 0.024).

^∗^ *p* < 0.05.

^∗∗^ *p* < 0.01.

### 3.3. Perceived PCC Climate of University Medical Center and Associated Demographic Variables

The overall mean PCQ‐P score was 5.00 (SD = 0.98) on a 1–6 scale. Among the three PCQ‐P subscales, Safety had the highest mean score (*M* = 5.24, SD = 0.99), whereas everydayness had the lowest (*M* = 4.75, SD = 1.14). At the item level, “A place where I rely on receiving the best care” had the highest mean score (*M* = 5.30), while “A place where I have choices, for example, what to wear” had the lowest (*M* = 4.55) (Supporting Table 2).

The bivariate analyses showed that religious affiliation was significantly associated with perceptions of the PCC climate (*H* = 8.11, *p* = 0.017), with nontheistic respondents reporting lower scores than Muslim and Christian participants. Kazakh language proficiency was positively associated with PCC climate (*ρ* = 0.13, *p* = 0.033), whereas Russian language proficiency was not significantly related (*ρ* = −0.01, *p* = 0.842). Full results of these analyses are presented in Supporting Table 4.

### 3.4. QOL Among the Patients

The physical health domain had a mean of 67.03 (SD = 14.38), which was lower relative to other domains. The psychological health domain had the highest mean at 80.03 (SD = 12.40). The social relationships domain recorded an average of 79.02 (SD = 15.94), while the environment domain achieved a mean of 77.29 (SD = 15.30). In terms of overall QOL, no respondents rated their QOL as very poor, while 0.4% rated it as poor, 7.5% as neither poor nor good, 53.7% as good, and 38.4% as very good. For health satisfaction, no respondents reported being very dissatisfied, while 5.5% were dissatisfied, 12.9% were neither satisfied nor dissatisfied, 57.3% were satisfied, and 24.3% were very satisfied (Supporting Table 3).

### 3.5. Relationship Between QOL and PCC

The overall P‐CIS score was positively associated with all WHOQOL‐BREF domains, including physical health (*ρ* = 0.23, *p* < 0.001), psychological health (*ρ* = 0.24, *p* < 0.001), social relationships (*ρ* = 0.25, *p* < 0.001), and environment (*ρ* = 0.29, *p* < 0.001). The personalization dimension was also positively associated with all QOL domains, with correlation coefficients ranging from *ρ* = 0.20 for physical health (*p* = 0.002) to *ρ* = 0.33 for psychological health (*p* < 0.001).

Similarly, the overall PCQ‐P score was positively associated with physical health (*ρ* = 0.24, *p* < 0.001), psychological health (*ρ* = 0.27, *p* < 0.001), social relationships (*ρ* = 0.25, *p* < 0.001), and environment (*ρ* = 0.28, *p* < 0.001). The hospitality, safety, and everydayness dimensions were also positively associated with each WHOQOL‐BREF domain (Table 4). These dimension‐level associations were examined only in the bivariate analyses and were not entered separately into the multivariable regression models.

**TABLE 4:** Bivariate correlations between quality of life and person‐centered care measures (*n* = 255).

Variable	Physical domain		Psychological domain		Social relationships		Environmental domain	
	*ρ*	*p*	*ρ*	*p*	*ρ*	*p*	*ρ*	*p*
Patient‐Centered Inpatient Scale overall mean score	0.23	< 0.001^∗∗∗^	0.24	< 0.001^∗∗∗^	0.25	< 0.001^∗∗∗^	0.29	< 0.001^∗∗∗^
Personalization	0.20	0.002^∗∗^	0.33	< 0.001^∗∗∗^	0.29	< 0.001^∗∗∗^	0.29	< 0.001^∗∗∗^
Empowerment	0.22	< 0.001^∗∗∗^	0.27	< 0.001^∗∗∗^	0.19	0.002^∗∗^	0.25	< 0.001^∗∗∗^
Information	0.07	0.304	0.05	0.472	0.15	0.019^∗^	0.18	0.004^∗∗^
Approachability/availability	0.21	0.001^∗∗^	0.09	0.162	0.14	0.022^∗^	0.20	0.001^∗∗^
Respectfulness	0.11	0.088	0.07	0.262	0.07	0.260	0.12	0.063
Miscellaneous	0.13	0.033^∗^	0.27	< 0.001^∗∗∗^	0.24	< 0.001^∗∗∗^	0.26	< 0.001^∗∗∗^
Patient‐centered	0.24	< 0.001^∗∗∗^	0.27	< 0.001^∗∗∗^	0.25	< 0.001^∗∗∗^	0.28	< 0.001^∗∗∗^
Climate questionnaire Overall mean score								
Hospitality	0.22	< 0.05^∗^	0.25	< 0.001^∗∗∗^	0.23	< 0.001^∗∗∗^	0.25	< 0.001^∗∗∗^
Safety	0.23	< 0.001^∗∗∗^	0.27	< 0.001^∗∗∗^	0.25	< 0.001^∗∗∗^	0.21	0.001^∗∗^
Everydayness	0.20	0.001^∗∗^	0.23	< 0.001^∗∗∗^	0.21	0.001^∗∗^	0.30	< 0.001^∗∗∗^

^∗^ *p* < 0.05.

^∗∗^ *p* < 0.01.

^∗∗∗^ *p* < 0.001.

### 3.6. Regression Analyses of Quality‐of‐Life Domains

Four multivariable linear regression models were estimated using the overall PCC experience and PCC climate scores rather than their individual dimensions. All four models were statistically significant: physical health, F(21, 233) = 3.05, *p* < 0.001, *R* ^2^ = 0.215, adjusted *R* ^2^ = 0.145; psychological health, F(21, 233) = 2.64, *p* < 0.001, *R* ^2^ = 0.192, adjusted *R* ^2^ = 0.120; social relationships, F(21, 233) = 3.34, *p* < 0.001, *R* ^2^ = 0.231, adjusted *R* ^2^ = 0.162; and environment, F(21, 233) = 5.41, *p* < 0.001, *R* ^2^ = 0.328, adjusted *R* ^2^ = 0.267. Thus, the adjusted models accounted for approximately 12.0%–26.7% of the variance across the four QOL domains (Table 1).

Kazakh language proficiency was positively associated with physical health (*B* = 1.12, *p* = 0.007), psychological health (*B* = 1.05, *p* = 0.004), social relationships (*B* = 1.48, *p* = 0.001), and environment (*B* = 1.49, *p* < 0.001). Overall PCC climate was positively associated with physical health (*B* = 2.00, *p* = 0.035) and psychological health (*B* = 1.90, *p* = 0.022). Overall PCC experience was positively associated with psychological health (*B* = 3.27, *p* = 0.047), social relationships (*B* = 4.67, *p* = 0.024), and environment (*B* = 3.82, *p* = 0.039).

Older age was associated with lower physical health (*B* = −0.31, *p* = 0.001) and social relationships scores (*B* = −0.25, *p* = 0.012). Compared to Muslim participants, Christian participants had higher social relationships scores (*B* = 10.54, *p* = 0.021). Compared to married participants, those categorized as cohabiting, divorced, or widowed had lower social relationships (*B* = −8.45, *p* = 0.008) and environment scores (*B* = −5.71, *p* = 0.044). Urban residence was positively associated with environment scores (*B* = 6.02, *p* = 0.009), as was an income of ≥ 750,000 KZT relative to the reference income category (*B* = 7.21, *p* = 0.044).

## 4. Discussion

This study examined the associations between adult inpatients’ perceptions of PCC, the broader PCC climate, and QOL during hospitalization. Overall PCC experience and PCC climate were associated with selected QOL domains after adjustment, although the models explained only part of the variation in QOL. PCC may therefore represent one element of patients’ QOL during hospitalization, alongside wider clinical, social, and contextual influences. At the dimension level, empowerment, perceived safety, and information provision were associated with particular QOL domains in the bivariate analyses. The pattern of findings also points to the importance of communication, patient involvement, perceived safety, and culturally responsive care, while recognizing that patients’ QOL reflects influences extending beyond PCC and the immediate care environment.

Patients reported generally positive PCC experiences, with empowerment receiving the highest score among the PCC dimensions. This suggests that many patients felt respected and involved in their care. This finding is consistent with self‐determination theory, particularly the importance of autonomy in supporting well‐being [14]. It also agrees with Alsubahi et al. [24], who found that patients with diabetes who reported higher PCC also reported greater satisfaction. Morris et al. [25] similarly showed that PCC‐related indicators were linked with emotional outcomes, although their findings also point to the importance of workplace and organizational context. In the present study, the high empowerment score should be interpreted alongside the lower rating for information provision. For nursing managers, this means that patient involvement should be supported not only through respectful interaction but also through clearer explanations, consistent communication, and opportunities for patients to ask questions and express preferences.

Participants also rated the hospital care climate positively, especially in relation to safety, respect, and support. This finding suggests that many patients perceived the inpatient environment as reliable and responsive. It is consistent with Al‐Sahli et al. [4], who reported favorable PCC‐climate perceptions among patients in Saudi hospitals. However, it differs from Ali and Charkos [26], who found stronger PCC ratings in private than public hospitals in Ethiopia, suggesting that care climate may vary by setting, resources, and organizational culture. In the Kazakhstani context, where patient engagement in care is still developing, these findings point to the need to maintain safe and respectful care environments while strengthening communication, privacy, and staff responsiveness.

Next, most participants reported favorable overall QOL. This finding is noteworthy given that the sample comprised hospitalized adults. Compared to WHOQOL‐BREF studies conducted in Brazil [27], Ethiopia [28], and Romania [29], participants in the present study reported comparatively favorable QOL, whereas a South Korean study reported higher QOL scores [30]. These cross‐study differences should be interpreted cautiously because the studies varied in participant characteristics, clinical conditions, sampling approaches, and healthcare contexts. Several factors may partly explain the favorable QOL reported in the present study. Patients with severe illness, cognitive impairment, delirium, communication difficulties, or extreme fatigue or pain were excluded, which may have resulted in a sample with relatively better functional status and greater capacity to participate. Data were also collected close to discharge, when participants may have been more clinically stable or perceived improvements in their condition. Social desirability, gratitude toward healthcare staff, cultural response patterns, and convenience sampling may also have influenced responses. Within the present study, psychological QOL was relatively more favorable than physical QOL. This pattern may reflect differences in how participants experienced psychological and physical well‐being during hospitalization, although the available data do not allow the underlying reasons to be determined. Overall, these findings indicate that we should interpret inpatient QOL within a broader clinical, social, and organizational context rather than attributing it solely to PCC.

Several sociodemographic findings showed that PCC was not experienced equally across patient groups. Older patients reported less positive PCC perceptions and lower physical health scores. This pattern aligns with Hobden et al. [9], who noted that older adults may view delayed or fragmented care as less patient‐centered. It is also supported by Albougami et al. [31], who reported that functional decline and dependency can reduce patients’ sense of autonomy and involvement in care. In the present study, the evidence suggests that older inpatients may need more intentional communication, repeated explanations, and support to remain involved in decisions about their care.

Patients with fewer financial resources and those from rural areas also reported less favorable PCC experiences. This finding is broadly consistent with Langford et al. [32], who reported associations between sociodemographic characteristics and perceived quality of patient‐centered communication. It also echoes Peng et al. [33] and Zhou et al. [34], who linked rural residence with inequities in patient‐centered care. Marital status was also relevant, as married patients reported more positive PCC experiences than those who were cohabiting, divorced, or widowed. This may reflect the practical role of family support in communication and decision‐making, as suggested by Cincidda et al. [35] and Lee and Kim [36]. Kazakh language proficiency was associated with higher QOL scores and more positive perceptions of the care climate. Given that Kazakh is the state language of Kazakhstan [37], this finding may reflect the relevance of language‐concordant communication in healthcare encounters. However, this association should be interpreted cautiously. Kazakh proficiency may also function as a proxy for broader factors such as cultural integration, educational access, social identity, or familiarity with the healthcare system. Therefore, the finding should not be interpreted as evidence that language proficiency directly improves psychological well‐being or PCC experience.

Overall, PCC experience and PCC climate were positively associated with selected QOL domains, although the pattern of association varied across domains. PCC climate was associated with physical and psychological QOL, whereas PCC experience was associated with psychological, social, and environmental QOL. These findings are broadly consistent with international evidence linking PCC with trust, satisfaction, and positive patient experiences [5, 38]. However, the present study adds context‐specific evidence from Kazakhstan, where previous research has focused largely on patient satisfaction, healthcare experience, and primary healthcare performance [12, 13]. By examining perceived PCC, care climate, and multidimensional QOL concurrently in specialized inpatient settings, this study extends the existing Kazakhstan literature beyond satisfaction‐based outcomes.

At the bivariate level, individual dimensions such as empowerment, safety, and information provision were associated with particular QOL domains, whereas the adjusted models identified associations with overall PCC experience and PCC climate alongside selected sociodemographic characteristics. This pattern broadly supports previous research emphasizing communication, patient involvement, perceived safety, and responsiveness as important features of person‐centered inpatient care [2, 5]. The findings may indicate that patients’ experiences of care reflect both interpersonal interactions and the broader organizational environment in which care is delivered. However, the dimension‐level findings were based on bivariate analyses only, and the exploratory nature of the analyses, together with the absence of adjustment for multiple comparisons, limits the strength of the conclusions that can be drawn. From a nursing management perspective, the findings suggest that communication, meaningful patient involvement, culturally and linguistically responsive care, and a care environment in which patients feel able to ask questions and express preferences warrant continued managerial attention [5, 32].

### 4.1. Limitations of the Study

Several limitations should be acknowledged. The cross‐sectional design limits causal interpretation of the relationships among PCC, PCC climate, and QOL outcomes. Longitudinal studies are needed to examine whether changes in PCC practices or care climate are followed by changes in patient‐reported outcomes over time. Common method bias is also an important limitation. All main variables, including PCC, care climate, and QOL, were measured using self‐report questionnaires completed by the same respondents at a single time point. This may have increased the risk of common method variance and inflated the observed associations. This concern is particularly relevant because PCC, care climate, and subjective QOL are conceptually related patient‐reported constructs. Although anonymity, voluntary participation, and independent questionnaire completion were used to reduce response pressure, future studies should consider additional strategies, such as collecting data from multiple sources, separating measurement time points, or including objective clinical or organizational indicators.

A further limitation is the issue of multiplicity. Because multiple associations were examined across the four QOL domains without adjustment for multiple comparisons, some findings, particularly those with *p* values close to 0.05, may represent chance associations. These findings should therefore be interpreted cautiously and replicated in larger studies before firm conclusions are drawn. The high QOL ratings should also be interpreted cautiously. Although 92.1% of participants rated their overall QOL as good or very satisfactory, the results may partly reflect response bias, social desirability, selective participation, or cultural response tendencies. The exclusion of patients with severe illness, cognitive impairment, delirium, communication disorders, or extreme fatigue or pain may also have contributed to higher QOL ratings by limiting participation to patients who were stable enough to complete the survey. In addition, because data were collected near discharge, patients may have reported more positive QOL due to perceived recovery, relief, or satisfaction with leaving the hospital. Future studies should include patients with a wider range of illness severity and consider triangulating self‐reported QOL with clinical indicators or qualitative data.

The adjusted regression models explained only a modest proportion of the variation in QOL, indicating that important determinants were not captured in the present analyses. Clinical and contextual factors such as illness severity, diagnosis, length of hospitalization, previous healthcare experiences, family support, and broader organizational conditions were not assessed and may have contributed to the observed QOL differences. Their omission may also have resulted in residual confounding. Future studies should incorporate a wider range of clinical, social, and organizational variables to better characterize the factors associated with QOL among hospitalized patients. The interpretation of language proficiency also requires caution. Kazakh language proficiency may reflect not only communication ability but also broader factors such as cultural integration, educational access, social identity, and familiarity with healthcare services. Future studies should examine these factors separately to clarify how language and culture relate to PCC perceptions and QOL.

The use of convenience sampling may also limit the generalizability of the findings beyond the study sample. In addition, the study examined only sociodemographic factors and did not assess other variables that may shape patients’ perceptions of PCC, such as illness severity, cultural beliefs, values, or norms. Finally, because the study was conducted in inpatient settings, the findings may not be directly applicable to other healthcare contexts, including outpatient clinics and primary care facilities.

## 5. Conclusions

Overall PCC experience and PCC climate were associated with selected QOL domains among hospitalized adults in Kazakhstan. Interpreted through self‐determination theory, the findings align with the view that autonomy, competence, and relatedness are relevant to patients’ experiences of well‐being. Associations involving individual PCC and care‐climate dimensions were observed only in the bivariate analyses, while the adjusted models focused on overall PCC experience and PCC climate. For nursing management, the results reinforce the importance of clear communication, meaningful patient involvement, culturally and linguistically responsive care, and a supportive inpatient environment. At the same time, patients’ QOL is likely influenced by a broader range of clinical, social, and organizational factors beyond PCC alone.

## 6. Implications for Nursing Management

The results have essential relevance for nursing management in inpatient settings. Although empowerment was associated with QOL only in the bivariate analysis, the adjusted models also showed associations between overall PCC measures and selected QOL domains. This suggests that relational aspects of care remain important to patients’ experiences, even though they represent only part of the wider influences on QOL. Nurse managers can strengthen this area by promoting communication practices that support shared understanding, patient autonomy, and meaningful involvement in care. Staff development plays a crucial role, especially since educational interventions have demonstrated their effectiveness in enhancing nurses’ competence in PCC [39].

The comparatively lower ratings for information provision also indicate an area for managerial attention. Clear and consistent communication about patients’ conditions, treatment options, and care processes may help reduce uncertainty and support greater involvement in care. Practical approaches could include brief communication check‐ins, teach‐back techniques, and culturally adapted patient information materials.

The associations involving language proficiency and socioeconomic characteristics further highlight the importance of culturally and linguistically responsive care. These findings should be interpreted cautiously, as language proficiency may also reflect broader cultural, educational, social, and healthcare‐related factors. Nursing leaders can nevertheless support staff through training, appropriate communication resources, and organizational practices that accommodate different linguistic needs and expectations of care.

Finally, the associations between PCC, care climate, and selected QOL domains support attention to the organizational conditions in which PCC is delivered. Nursing leaders may consider strategies that protect time for meaningful patient interaction, strengthen interprofessional collaboration, and embed patient involvement within routine care processes. Such approaches may help create care environments that are more consistently responsive to patients’ needs while supporting the quality and equity of inpatient care.

## Author Contributions

Conceptualization: Amirshair Abdilda, Joseph Almazan, and Jonas Preposi Cruz. Methodology: Amirshair Abdilda, Joseph Almazan, and Jonas Preposi Cruz. Data curation: Amirshair Abdilda and Gulnur Nadirbekova. Formal analysis: Amirshair Abdilda and Jonas Preposi Cruz. Investigation: Amirshair Abdilda and Gulnur Nadirbekova. Writing–original draft: Amirshair Abdilda, Joseph Almazan, and Jonas Preposi Cruz. Writing–review and editing: all authors. Supervision: Joseph Almazan and Jonas Preposi Cruz. Project administration: Amirshair Abdilda.

## Funding

This Open Access publication was supported by Nazarbayev University under the transformative agreement with Wiley.

## Disclosure

All authors approved the final manuscript and agree to be accountable for all aspects of the work.

Institutional Affiliations Note. Sigma Theta Tau International: Jonas Preposi Cruz–486 Upsilon Xi Chapter, Swansea University, Wales, UK.

## Ethics Statement

Ethical approval for this study was obtained from the Nazarbayev University Institutional Research Ethics Committee (NU‐IREC; Application No. 986/05112024; approved 27 January 2025) and the Local Bioethics Committee of the Corporate Fund University Medical Center (Ref. No. 2025/03‐004; approval date pending verification from the official UMC approval letter). All participants provided written informed consent before data collection.

## Consent

Please see the Ethics Statement.

## Conflicts of Interest

The authors declare no conflicts of interest.

## General Statement

Person‐centred care: https://www.safetyandquality.gov.au/our-work/partnering-consumers/person-centred-care. Person‐Centred Care: https://www.hee.nhs.uk/our-work/person-centred-care. WHOQOL: Measuring Quality of Life: https://www.who.int/tools/whoqol.

## Supporting Information

Additional supporting information can be found online in the Supporting Information section.

## Supporting information


**Supporting Information 1** Supplementary Table 1. Results of the descriptive analysis on the Patient‐Centered Inpatient Scale (*n* = 255). Supplementary Table 2. Results of the descriptive analysis on the Patient‐Centered Climate Questionnaire (*n* = 255). Supplementary Table 3. Results of the descriptive analysis on the World Health Organization Quality of Life–BREF (*n* = 255). Supplementary Table 4. Results of the bivariate analyses between the respondents’ demographic variables and PCC climate of the hospital (*n* = 255).

**Supporting Information 2** STROBE checklist cross‐sectional.

## Data Availability

The data supporting this study are available from the corresponding author upon reasonable request. Restrictions apply due to privacy/ethical considerations.
