# Supplementary material for: Person‐Centered Care, Care Climate, and Quality of Life: Evidence From Inpatient Settings in Kazakhstan
Source: J Nurs Manag. 2026 Oct 21;2026:2533736. doi: 10.1155/jonm/2533736 (PMC13686390; doi:10.1155/jonm/2533736)
Supplement: Supplementary file 1 [file JONM-2026-2533736-s001.pdf]

Supplementary Table 1
Results of the Descriptive Analysis on the Patient-Centered Inpatient Scale (n = 255)

Variable	Minimum	Maximum	Mean	SD
Personalization	1.75	5.00	3.92	0.64
The doctors understood fully what I was going through	1.00	5.00	4.40	0.89
I was made to feel at home very quickly	1.00	5.00	4.13	0.94
By the time I left I felt the staff were my friends not just staff members	1.00	5.00	4.00	1.02
The service was designed more for the convenience of staff than for patients	1.00	5.00	3.15	1.31
Empowerment	1.00	5.00	4.09	0.66
I felt as though the staff and I were partners in the whole process of my care	1.00	5.00	4.01	1.11
Nobody asked me what I thought about my treatment	1.00	5.00	3.94	1.01
The nurses didn't seem to listen to what I said	1.00	5.00	4.14	0.88
The nurses always listened attentively to what I said	1.00	5.00	4.27	1.00
Information	1.00	5.00	3.44	0.84
At times I felt the doctors did not want me to ask questions	1.00	5.00	3.95	1.05
I wish the doctor had given me a fuller explanation of my condition	1.00	5.00	2.67	1.36
I was sometimes left waiting, not knowing what was going on	1.00	5.00	3.81	1.00
The doctors should have given me more information about my treatment	1.00	5.00	3.31	1.22
Approachability/ Availability	1.00	5.00	3.62	0.81
I was often unable to locate nurses for assistance	1.00	5.00	4.14	0.90
Sometimes I felt a bit abandoned by the staff while in hospital	1.00	5.00	4.19	0.83
I would have liked to speak with the nurses more often	1.00	5.00	3.15	1.22
I would have liked to speak to the doctors more often	1.00	5.00	2.99	1.29
Respectfulness	1.00	5.00	3.98	0.84
Sometimes staff stood near me talking about me as if I wasn't there	1.00	5.00	4.20	0.84

The staff were very concerned about my privacy	1.00	5.00	3.77	1.25
Miscellaneous	1.00	5.00	4.07	0.93
I had plenty of choice in the food they provided	1.00	5.00	3.84	1.16
While in hospital I was waited on hand and foot	1.00	5.00	4.29	0.97
Patient-Centered Inpatient Scale Overall Mean	2.30	5.00	3.82	0.52

Supplementary Table 2

Results of the Descriptive Analysis on the Patient-Centered Climate Questionnaire (n = 255)

Variable	Minimum	Maximum	Mean	SD
Hospitality	1.00	6.00	4.97	1.00
A place where the staff is knowledgeable.	1.00	6.00	5.20	1.11
A place where I feel welcome.	1.00	6.00	5.00	1.21
A place where it is easy to talk to the staff.	1.00	6.00	5.16	1.03
A place where the staff take notice of what I say.	1.00	6.00	5.11	1.13
A place where the staff use language I can understand.	1.00	6.00	5.20	1.14
A place where the staff make a little extra effort on my behalf.	1.00	6.00	4.83	1.30
A place where I have choices, for example, what to wear.	1.00	6.00	4.55	1.44
A place where I can get "that little bit extra."	1.00	6.00	4.71	1.37
Safety	1.00	6.00	5.24	0.99
A place where I rely on receiving the best care.	1.00	6.00	5.30	1.05
A place where I feel in safe hands.	1.00	6.00	5.29	1.08
A place where the staff come quickly when I need help.	1.00	6.00	5.18	1.11
A place which is neat and clean.	1.00	6.00	5.27	1.12
A place where the staff have time for the patients.	1.00	6.00	5.19	1.11
Everydayness	1.00	6.00	4.75	1.14
A place where there is something nice to look at.	1.00	6.00	4.76	1.28
A place which feels homely even though I am in an institution.	1.00	6.00	4.75	1.30
A place where it is possible to get unpleasant thoughts out of your head.	1.00	6.00	4.74	1.27
A place where people talk about ordinary things, not just illness.	1.00	6.00	4.75	1.26
Overall Mean	1.00	6.00	5.00	0.98

Supplementary Table 3

Results of the Descriptive Analysis on the World Health Organization Quality of Life - BREF (n = 255)

Variable	Minimum	Maximum	Mean	SD
Quality of life dimensions				
Physical health domain	17.86	100.00	67.03	14.38
Psychological health domain	37.50	100.00	80.03	12.40
Social relationships domain	16.67	100.00	79.02	15.94
Environment domain	28.13	100.00	77.29	15.30
General perception of quality of life	n	%		
Very poor	0.0	0.0		
Poor	1	0.4		
Neither	19	7.5		
Good	137	53.7		
Very good	98	38.4		
General satisfaction on health				
Very dissatisfied	0.0	0.0		
Dissatisfied	14	5.5		
Neither	33	12.9		
Satisfied	146	57.3		
Very satisfied	62	24.3		

Supplementary Table 4

Results of the Bivariate Analyses between the Respondents' Demographic Variables and Person-Centered Care Climate of the Hospital (n = 255)

Variable	Mean Rank	Statistical Test	*p*
Age		*rho* = 0.00	.997
Gender			
Male	125.99	*U* = 7523.00	.728
Female	129.28		
Religion			
Islam	131.35	*H* = 8.11	.017*
Christianity	131.75		
Non-theistic (Atheist, Agnostic, no religion)	77.28		
Hospital			
Hospital A	124.84	*U* = 7356.50	.591
Hospital B	129.94		
Area of residence			
Rural area	115.70	*U* = 4510.00	.187
Urban area	131.00		
Education		*rho* = -0.01	.942
Occupation			
Employed	123.40	*H* = 1.66	.647
Unemployed	133.24		
Self-employed	138.74		
Retired	133.81		
Marital status			
Single	125.83	*H* = 0.19	.911
Married	128.90		
Others (Cohabiting, divorced, widowed)[a]	122.76		
Financial status		*rho* = 0.05	.399
Kazakh language proficiency		*rho* = 0.13	.033*
Russian language proficiency		*rho* = -0.01	.842

Note. *Significant at .05 level
